# Point-of-Care Diagnostics (POCD) in Resource-Limited Settings

**DOI:** 10.3390/diagnostics14171926

**Published:** 2024-08-31

**Authors:** Paul K. Drain

**Affiliations:** Departments of Global Health, Medicine, and Epidemiology, University of Washington, Seattle, WA 98195, USA; pkdrain@uw.edu

## 1. Introduction

Diagnostic testing is critical to provide adequate healthcare, and the emergence of various rapid point-of-care diagnostics (POCD) allows for greater access and broader implementation in resource-limited settings [1]. Since many high-technology solutions are either unaffordable or unfit for use in some global settings, various solutions for developing low-cost, portable, and user-friendly diagnostics are needed [2]. The key characteristics of various POCD tests have been described [3,4]. Since then, the role and value of diagnostics, including POCD, for various infectious and non-communicable diseases have been established [5].

However, one common prerequisite for developing POCDs is proper knowledge of the operational challenges, technical limitations, and user needs within intended settings of use [6]. POCD will need to completement the development and expansion of lab-based testing programs [7,8]. To meet these needs, the World Health Organization (WHO) has established criteria for diagnostic prequalification (approval) [9], which is a necessary status for countries to purchase in vitro diagnostics using multilateral funding. In 2023, the WHO and partners have updated an Essential Diagnostics List to simplify the planning and procurement processes [10]. Overall, there has been a rapid expansion in both the quantity and quality of diagnostic tests and testing, and more innovation with the integration of machine learning and artificial intelligence (AI) will be coming soon.

In this Special Issue on “Point-of-Care Diagnostics (POCD) in Resource-Limited Settings”, we highlight six manuscripts that have given careful consideration to the various technological specifications and implementation science aspects to make POCDs successful for improving health outcomes in various resource-limited settings.

## 2. An Overview of Published Articles

Arriaga-Cázares and colleagues (Contribution 1) addressed the issues of dyslipidemia in childhood that, if left undiagnosed and untreated, can lead to atherosclerotic cardiovascular disease in adulthood, which remains a leading global cause of mortality. The research team sought to evaluate the impact of screening cholesterol levels using a rapid point-of-care device among children in a family practice setting in Mexico. They considered whether a targeted screening approach may be a more practical approach than a universal screening approach among a cohort of 183 children. The results showed that testing among children with a family history of cholesterolemia and/or identifiable risk factors had a higher prevalence of children screening positive for dyslipidemia. Furthermore, the cholesterol screening was successfully performed in the clinic using a device (Accutrend Plus, Roche Diagnostics) that provided results within three minutes. Overall, this study suggests that targeted screening for dyslipidemia can be performed using POCDs among children at higher risk in a family practice clinic.

The work by Jang and colleagues (Contribution 2) evaluated a novel, rapid diagnostic tool for measuring CD4+ T lymphocytes in blood, which is an important measurement for people living with HIV (PLWHIV) or other immunodeficiencies. The Microscanner Plus is a simple, automated image-based cell counter that is smaller and less expensive than a flow cytometer machine and can be used at the clinical point of care. The team evaluated the device among 134 people, 87 of whom were PLWHIV, in South Korea. The Microscanner Plus had a lower limit of quantification, at 15.3 cells/microliter of whole blood. When compared to flow cytometry, the Microscanner Plus had a high correlation coefficient (R = 0.99), and showed consistent and reliable results for measuring CD4+ T lymphocytes. Overall, the Microscanner Plus, which tests whole blood, demonstrated high accuracy when compared to gold-standard flow cytometry testing. This device may be used in clinics for monitoring PLWHIV or testing for other immunodeficiencies in resource-limited settings.

Nakyanzi and colleagues (Contribution 3) conducted a qualitative study to understand the acceptability of point-of-care HIV viral load testing among pregnant and postpartum women living with HIV (PWLHIV) in Uganda. This study is a subset of a randomized controlled trial to evaluate whether clinic-based, point-of-care HIV viral load testing during pregnancy and infant testing at delivery was associated with improved maternal viral suppression after delivery. In this study, 22 PWLHIV who were part of the point-of-care testing arm took part in focused, in-depth interviews. Several themes emerged from these interviews, including that point-of-care viral load testing motivated women to remain adherent to their HIV medications. In turn, PLWHIV suggested that testing helped them protect their newborn from HIV and also improved their emotional well-being. The authors concluded that POC HIV viral load testing could be offered to PWLHIV in high HIV burden settings. This study also highlights several secondary benefits that can occur by using POCD to provide actionable information to patients in a timely manner.

In a study by Bărbulescu and colleagues (Contribution 4), fecal occult blood testing (FOBT), which is available as a simple, rapid POCD, was used to develop a scoring method for colorectal cancer screening. The team developed a cohort of 112 patients over 40 years of age in Romania and collected data for various risk factors, including age, sex, body mass index, smoking, diabetes, dyslipidemia, and hypertension. The analyses focused on identifying the risk factors that lead to having a positive FOBT, which can indicate a need for further testing. The analyses suggested that being older (>60 years), male, smoker, obese, and with cardiovascular risk factors was the highest risk profile for having a positive FOBT. The team further developed a score point allocation for assessing risk. While screening for colorectal cancer is valuable for everyone over 50 years old, this study further identifies the subset of people who may be at greatest risk for having a positive FOBT screening test for colorectal cancer.

Takenaka and colleagues (Contribution 5) developed a rapid test for C-reactive protein (CRP), a general inflammatory biomarker, and a rapid test for providing differential cell counts of leukocytes (HemoCue WBC DIFF). The CRP test was a simple lateral flow assay test that provided semi-quantitative measurements after just five minutes. The study was conducted among 31 patients admitted to a hospital with community-acquired pneumonia in Niigata, Japan. Overall, both the rapid CRP test and the HemoCue WBC DIFF test had good agreement with the laboratory-based testing methods. The team did observe a limited correlation for the neutrophil-to-lymphocyte ratio for the rapid test. Overall, both POCD were considered to have met the standards for possible use in decentralized, resource-limited settings.

The work by Sagoe and colleagues (Contribution 6) describes the development of a novel, rapid test for SARS-CoV-2 infection. The team used a hybridization chain reaction (HCR) and a CRISPR/Cas12a complex to detect the nucleocapsid gene. In addition, the results of the assay were visualized using a lateral flow assay design with CRISPR/Cas12a. The team then tested their novel assay on five positive and five negative nasopharyngeal specimens from adults in Thika, Kenya. For clinical validation, each of the five positive specimens showed a positive hybridization on the assay, while all five negative specimens did not show a reaction. The team also conducted additional testing among other pandemic viral infections to demonstrate the high specificity of the assay. Overall, the team was successful for a pilot demonstration of using HCR and CRISPR/Cas12a to develop another POCD for SARS-CoV-2.

## 3. Conclusions

Collectively, these studies highlight the range and need for POCDs in resource-limited settings. Some of these manuscripts focused on the development of novel POCDs, and others focused on evaluating manufactured POCDs in clinic- or community-based settings. Other manuscripts have highlighted the need for understanding efficient use of POCDs, and one manuscript highlighted the additional psychological benefits that may be gained through the use of POCDs. This series is perhaps a start in the ever-expanding development, evaluation, and implementation of POCDs for both infectious and non-infectious indications in resource-limited settings.
